# Thermal Pyocyanin Sensor Based on Molecularly Imprinted Polymers for the Indirect Detection of *Pseudomonas aeruginosa*

**DOI:** 10.1021/acssensors.2c02345

**Published:** 2023-01-04

**Authors:** Margaux Frigoli, Joseph W. Lowdon, Manlio Caldara, Rocio Arreguin-Campos, Julia Sewall, Thomas J. Cleij, Hanne Diliën, Kasper Eersels, Bart van Grinsven

**Affiliations:** Sensor Engineering Department, Faculty of Science and Engineering, Maastricht University, P.O. Box 616, 6200 MDMaastricht, The Netherlands

**Keywords:** Pseudomonas aeruginosa detection, molecularly imprinted polymer (MIP), infection control, pyocyanin, heat-transfer method (HTM), MIP-based sensor, bacterial analysis

## Abstract

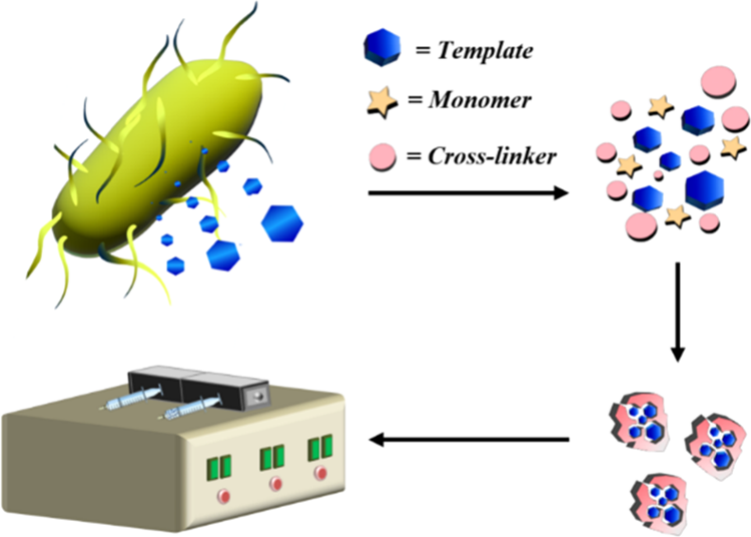

*Pseudomonas aeruginosa* is a ubiquitous multi-drug-resistant bacterium, capable of causing serious illnesses and infections. This research focuses on the development of a thermal sensor for the indirect detection of *P. aeruginosa* infection using molecularly imprinted polymers (MIPs). This was achieved by developing MIPs for the detection of pyocyanin, the main toxin secreted by *P. aeruginosa*. To this end, phenazine was used as a dummy template, evaluating several polymeric compositions to achieve a selective MIP for pyocyanin recognition. The sensitivity of the synthesized MIPs was investigated by UV–vis analysis, with the best composition having a maximum rebinding capacity of 30 μmol g^–1^ and an imprinting factor (IF) of 1.59. Subsequently, the MIP particles were immobilized onto planar aluminum chips using an adhesive layer, to perform thermal resistance measurements at clinically relevant concentrations of pyocyanin (1.4–9.8 μM), achieving a limit of detection (LoD) of 0.347 ± 0.027 μM. The selectivity of the sensor was also scrutinized by subjecting the receptor to potential interferents. Furthermore, the rebinding was demonstrated in King’s A medium, highlighting the potential of the sensor for the indirect detection of *P. aeruginosa* in complex fluids. The research culminates in the demonstration of the MIP-based sensor’s applicability for clinical diagnosis. To achieve this goal, an experiment was performed in which the sensor was exposed to pyocyanin-spiked saliva samples, achieving a limit of detection of 0.569 ± 0.063 μM and demonstrating that this technology is suitable to detect the presence of the toxin even at the very first stage of its production.

*Pseudomonas aeruginosa* is a Gram-negative opportunistic bacterium commonly found in soil, water, and vegetation.^1^ This multi-drug-resistant microorganism can proliferate in different types of environments as it can exploit a multitude of sources for nourishment.^2^ Due to its aggressive infectious properties, *P. aeruginosa* was inserted in the priority list of multi-drug-resistant bacteria issued by the WHO in 2017.^3^*P. aeruginosa* can cause different types of medical conditions (e.g., skin, ear, ocular, and lung infections) and is particularly dangerous for immunosuppressed patients,^4^ with one of the most minacious infections affecting people ailed by Cystic Fibrosis.^5^ In 2020, more than 40,000 children and adults were affected by this disease in the United States, and it has been evaluated that more than 60% of them were infected with *P. aeruginosa*.^6,7^ As a consequence of its danger and ubiquity, reliable and rapid detection of *P. aeruginosa* infections is crucial for the diagnosis and treatment of patients. Therefore, a vast repertoire of analysis methodologies aimed at detecting the bacterium has been developed over the years.^8^

The different technologies currently available for *P. aeruginosa* detection can be divided into two main categories: (a) methods based on the detection of the whole microorganism and (b) methods based on the detection of bacterial metabolites. The first category includes a variety of microbiological methods such as pseudomonas isolation agar (PIA), polymerase chain reaction (PCR), enzyme-linked immunosorbent assay (ELISA), or immunochromatographic assay (ICA). These technologies are characterized by their high reliability and sensitivity, but they are also expensive and require qualified personnel. Alternatively, multiple low-cost biosensing methods have been developed for the detection of bacteria. Typically, these platforms are based on antibodies or enzymes coupled to electrochemical or optical transducers. These sensors are suitable for point-of-care applications as they are cheaper and more user-friendly, but they require strict control over the working conditions (pH, temperature, pressure) because of the nature of the biological recognition elements used.^9^

Methods belonging to the second category exploit the large variety of metabolites secreted by *P. aeruginosa* itself, including molecules regulating quorum sensing (autoinducers), biofilm formation components, or virulence factors.^10,11^ Among the procedures that detect these compounds, HPLC/MS, UV–vis spectroscopy, fluorescence-based, or electrochemical methods are the most frequently encountered.^12,13^ However, similarly to the previously mentioned methods for whole bacteria detection, these methods are very sensitive but necessitate expensive equipment that requires trained personnel and transport of the sample to a specialized lab.^14^ Ideally, a technology should be able to quickly identify a *P. aeruginosa* infection directly at the point of care. In this way, the patient can be immediately treated with the appropriate medication, significantly improving the disease prognosis. However, this type of technology is still largely missing in current healthcare practices. Therefore, despite the effective and interesting technologies that currently exist, there still remains a need for alternative methods that can offer a fast, low-cost alternative that can be used directly by practitioners.

An emerging approach that matches these requirements is the development of sensing systems based on molecularly imprinted polymers (MIPs), chemical adducts synthesized to selectively detect a specific target.^15^ These receptors are polymeric materials presenting selective cavities that can specifically rebind the template through a morphological and functional complementarity, similar to the key-and-lock principle observed in biological interactions such as antibody–antigen and enzyme–substrate recognition. To prepare a MIP, the template is dissolved in a porogen together with a functional monomer, a cross-linker, and an initiator.^16^ The reagents used for MIP synthesis are carefully chosen, often aimed at maximizing noncovalent interaction between the functional groups of the template and the components of the polymeric matrix.^17−19^ The template species can then be removed by mechanical grinding and solvent-based extraction methods such as Soxhlet extraction, leaving vacant binding sites in the polymeric matrix.^20^ The procedures used to synthesize MIPs are numerous (precipitation, emulsion, solid-phase synthesis, and electropolymerization),^21−23^ though monolithic bulk free-radical polymerization remains the most straightforward approach.^24^ In addition to their low-cost and scalable production process, MIPs also offer a robust alternative to biological receptors that often require physiological conditions to operate optimally and have limited shelf-life. In fact, MIPs are stable under a wide range of temperatures, have a very long shelf-life, and are not degraded by organic solvents, acids, or bases.^25^ These specific features make MIPs interesting receptors for integration into sensors for point-of-care diagnostics. Thus, MIPs have been used in biomimetic sensing platforms for the detection of numerous bacterial metabolites by combining them with various transducer principles including electrochemical, acoustic wave, or optical readout devices.^26−28^

Although these biosensor platforms have proven to be very promising, they often require relatively specialized readout equipment and correct data interpretation can be complicated. To overcome this, a low-cost and straightforward alternative transducer principle, coined the heat-transfer method (HTM), was introduced by the authors in 2012.^29^ This sensing technique offers the possibility to study the selectivity and sensitivity of receptor layers (e.g., MIPs) for the desired target by simply studying the heat transport over a functional layer.^30−32^ The HTM has been successfully combined with synthetic receptors to detect a wide array of targets, ranging from small molecules to larger biological entities.^33−35^ Bacteria detection is a particularly interesting application, but it requires the use of bacteria cells as templates to create synthetic receptors.^36,37^ This limits the scalability of the receptor synthesis and increases the measurement cost significantly.

This work is therefore aimed at developing a sensor for indirectly detecting *P. aeruginosa* by targeting pyocyanin, one of the main toxins secreted by the bacterium.^38^ Due to the powerful radical scavenging capability of the pyocyanin making bulk free-radical polymerization cumbersome,^39^ a dummy imprinting approach was instead adopted using phenazine, a structural analogue, as a template (Figure 1).

**Figure 1:**
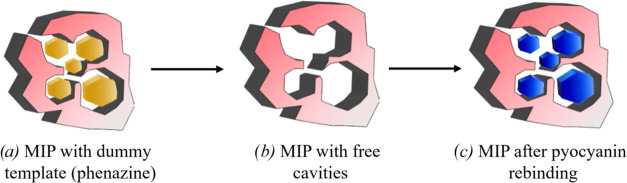
Dummy template technology representation. (a) Synthesis using phenazine as a dummy template; (b) free cavities formed after template extraction; and (c) pyocyanin detection after rebinding.

In this study, we optimized the MIP synthesis protocol for the specific recognition of pyocyanin by testing various MIP compositions and studying the resulting binding capacity and imprinting factors. The best MIPs were then integrated into an HTM-based sensor that was characterized in buffer solution to determine its linear range and limit of detection. The selectivity of the sensor was assessed in the presence of structural analogues and biologically relevant interferants, namely, phenazine methosulfate, glucose, ascorbic acid, and riboflavin (chemical structures of these compounds can be found in the Supporting Information, Chart S1).

To test the performance of the pyocyanin sensor, it was used for indirectly relaying the pyocyanin concentration as measured in King’s A medium to the concentration of *P. aeruginosa* in the same sample. In a final experiment, a first proof of application for medical diagnostics was explored, exposing the sensor to pyocyanin-spiked saliva samples. The results of this experiment, clearly illustrate that the sensor is capable of detecting pyocyanin in complex biological samples without the need for laborious techniques to extract the toxin.

## Materials and Methods

### Chemicals and Reagents

Phenazine methosulfate (>90%), tris hydrochloride (>99%), ethylene glycol dimethacrylate (98%), 2,2′-azobis(2-methylpropionitrile) (98%), (vinylbenzyl) trimethylammonium chloride (VBTMA) (99%), glycerol (>99.5%), ethanol (70%), l-ascorbic acid (99%), riboflavin (>98%), poly(vinyl chloride) (average *M*_w_ 80,000, average *M*_n_ 47,000), cetrimide agar, LB broth, proteose peptone, magnesium chloride hexahydrate (>99%), potassium sulfate (>99%), acetic acid (99%), and tetrahydrofuran (>99.9%) were purchased from Sigma-Aldrich. Phenazine (>99%) was purchased from Alfa Aesar. Divinylbenzene (80%, mixture of isomers), methanol (>99%), phenazine methosulfate (98%), and PBS tablets were purchased from Fisher Scientific. 1-Methoxyphenazine (>93%) and β-d-glucose (>85%) were purchased by TCI Chemicals. Hexane (>99%) and chloroform (>99%) were purchased from BioSolve. All of the aqueous solutions were prepared using deionized water purified with Stakpure Omnia Tap UV 12 L/h water system with a final water resistivity of 18.1 MΩ cm^–1^. Aluminum chips (0.5 mm thickness) were purchased from Conrad Electronic and cut to the final desired dimensions of 1 × 1 cm^2^. Poly(dimethylsiloxane) (PDMS) stamps were synthesized with a Sylgard 184 elastomer kit obtained from Mavom N.V. (Schelle, Belgium). *P. aeruginosa* (DSM 50071-0121-001) were purchased freeze-dried from DSMZ (Braunschweig, Germany) and reactivated following DSMZ procedure.^40^

### Pyocyanin Synthesis

A 100 mL (10 mM) solution of tris-HCl in water was prepared and then adjusted with NaOH (0.1 M) to reach a final pH of 7.4. Phenazine methosulfate (100 mg, 0.326 mmol) was added to the flask and stirred for 2.5 h at room temperature in the dark.^41^ Meanwhile, the flask was irradiated for the duration of the reaction with blue UV light using a BlueWave 75 UV Curing Spot Lamp purchased from Dymax. The reaction mixture was then transferred to a 1 L separation funnel and extracted with chloroform (3 × 200 mL). After removing the volatiles under reduced pressure, the obtained slug was suspended in a 4 mL mixture of H_2_O/Methanol (1:1) to prepare it for HPLC purification. The final chromatograms and gradients are shown in the Supporting Information (Figure S1 and Table S1), as well as the LC-MS analysis (performed using an LCMS-2020 purchased from Shimadzu, Figure S2) and ^1^H-NMR (performed using a 400 MHz Year Hold Superconducting Magnet, 400JJYH, purchased from Jeol Ltd., Figure S3) used to assess the product’s purity. The spectra and chromatograms obtained are in line with those found in previous works.^42,43^ The final product was obtained with >98% purity as a dark blue powder that could be stored dry, in water, or in chloroform for up to 30 days at −20 °C.

### Molecularly Imprinted Polymers Synthesis

To obtain a molecularly imprinted polymer (MIP) for pyocyanin detection a free-radical monolithic bulk polymerization approach was used, which has been reported for the synthesis of MIPs for other targets in previous work.^30,34,44^ Due to the radical scavenging properties of the envisioned target, a dummy template approach was selected. To achieve this, phenazine was chosen as a template to create pyocyanin receptors due to its structural similarities with pyocyanin. In addition, phenazine is not water-soluble and does not typically occur in the biological samples in question and will therefore not interfere with pyocyanin detection. To create the MIPs, phenazine (0.277 mmol, 1 equiv, 50 mg) was dissolved in 5 mL of chloroform inside a 10 mL glass vial, followed by the addition of the functional monomer and the cross-linker to form a pre-polymerization mixture. To optimize the MIP synthesis, various MIP compositions were studied using (vinylbenzyl) trimethylammonium chloride (VBTMA) and methacrylic acid (MAA) as monomers, and ethylene glycol dimethacrylate (EGDMA) and divinylbenzene (DVB) as cross-linkers as shown in Table 1. The pre-polymerization mixture was then sonicated for 5 min, after which the thermal initiator AIBN (0.554 mmol, 2 equiv, 91 mg) was added. The mixture was further sonicated a second time for 5 min, degassed with N_2_ for 10 min, and left to react overnight at 65 °C in an oil bath. The resulting imprinted polymer was crushed with a spatula, washed with methanol, and then dried in the oven overnight at 65 °C before milling it using Fritsch Planetary Micro Mill Pulverisette7 premium line (300 rpm, 5 min, 10 mm balls). After milling, two Soxhlet extraction cycles were performed to remove the template. The first extraction cycle consists of extraction using a mixture of methanol and acetic acid (9:1) performed overnight followed by a second extraction cycle in methanol. In between the two cycles, the MIP powder was again dried and milled to make the particles more homogeneous. As a negative control, a non-imprinted polymer (NIP) was synthesized for each composition using the same recipe used to synthesize the MIPs but without the addition of the template.

**Table 1:** Part of the MIPs Compositions Investigated^a^

	monomer	cross-linker	solvent	template	IF (At *C*_f_ = 0.15 mM)	*S*_b_ (μmol/g) (At *C*_f_ = 0.15 mM)
MIP01	VBTMA (1.66 mmol)	EGDMA (5.54 mmol)	CHCl_3_ (5 mL)	phenazine (0.277 mmol)	1.59	30
MIP02	VBTMA (1.66 mmol)	EGDMA (5.54 mmol)	DMSO (5 mL)	phenazine (0.277 mmol)	1.34	21
MIP03	VBTMA (1.1 mmol)	EGDMA (3.32 mmol)	CHCl_3_ (5 mL)	phenazine (0.277 mmol)	1	25
MIP04	MAA (1.66 mmol)	DVB (3.32 mmol)	DMSO (5 mL)	phenazine (0.277 mmol)	0.91	6
MIP05	MAA (2.77 mmol)	DVB (3.88 mmol)	CHCl_3_ (5 mL)	phenazine (0.277 mmol)	0.38	10
MIP06	VBTMA (2.77 mmol)	DVB (3.88 mmol)	DMSO (5 mL)	phenazine (0.277 mmol)	1.17	38
MIP07	MAA (1.66 mmol)	DVB (2.77 mmol)	DMSO (3 mL)	phenazine (0.277 mmol)	0.99	60

a For each batch, 0.554 mmol of AIBN was used as an initiator.

### Batch-Rebinding Experiments

The binding capacities of the different MIPs studied were assessed using batch-rebinding experiments using a UV–vis spectrophotometer (Shimadzu). To determine the binding isotherm for each of the MIPs, six samples of 20 mg were weighed inside 10 mL glass vials and suspended in 5 mL of aqueous solution containing an increasing concentration of pyocyanin at pH 7.4 (0.1–0.4 mM). After 90 min of agitation at room temperature, the samples were filtered using a 0.45 μm VWR filter and the filtrates were collected to perform UV–vis analysis. From the collected spectra, the remaining free concentration (*C*_f_) of pyocyanin could be determined as well as the amount of analyte bound to the MIP (*S*_b_). Subsequently, this data was used to construct binding isotherms for each of the MIPs under investigation. The same procedure was done using the NIPs as reference, enabling a direct comparison of pyocyanin binding at each concentration and the imprinting factor (IF) and binding capacity (*S*_b_) to be calculated.

### Aluminum Chip Preparation

After cutting the aluminum sheets in the desired dimension for the final sensor (1 cm × 1 cm × 0.005 cm), the obtained chips were coated with a PVC layer (200 mg of PVC in 5 mL of THF) using a spin coater (800 rpm, 60 s). These chips were put on a heating plate at 100 °C to soften the PVC layer above its glass-transition temperature to facilitate the particles’ permeation into the layer. A PDMS stamp of approximately 0.3 cm × 1 cm × 1 cm was used to trap the MIP/NIP particles and to thereafter stamp the particles onto the sticky adhesive-coated aluminum chips. The PDMS stamp was pressed against the chip for 1 min using a spatula, and the resulting sensor was washed with deionized water to remove any excess/weakly bound MIP/NIP powder.

### Sensing Setup

**Figure 2:**
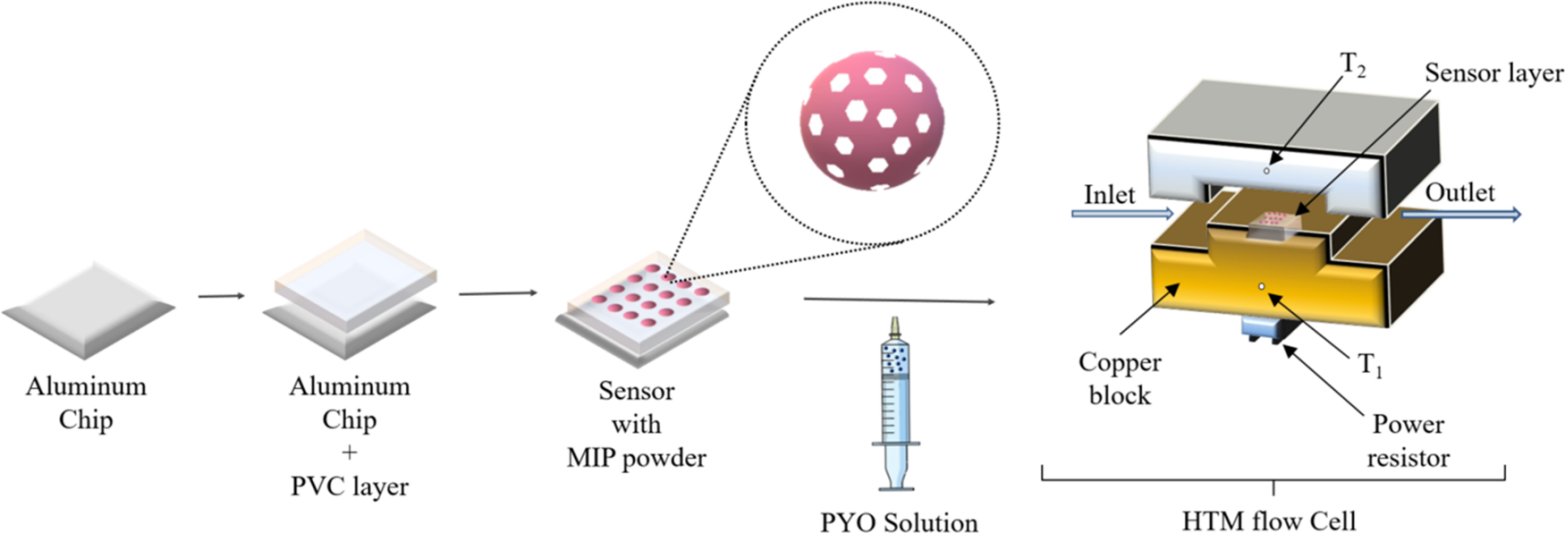
Samples preparation and flow cell illustration for HTM measurements.

After optimizing the synthesis procedure, MIPs made using the best recipe were used to further study rebinding with the heat-transfer method (HTM) in complex samples. The thermal transducer principle has been extensively studied for a wide range of targets, as described in previous works of our research group.^31,32,45^ In short, the HTM technology measures the heat transfer over a MIP-covered aluminum chip, positioned in between a copper block and a polycarbonate flow cell (*A* = 28 mm^2^, *V* = 110 μL). The function of the copper block is to transfer the heat generated by a power resistor, to the bottom side of the MIP-covered chips. The temperature of the copper block (*T*_1_) is controlled by a feedback loop consisting of a type K thermocouple (TC Direct, the Netherlands), a 22 Ω power resistor, and a software-driven Proportional-Integral-Derivative controller (software and data acquisition card LabView, National Instruments, Austin, TX, and data logger TC-08 by Pico Technology, Sint Neots, U.K.) with *P* = 10, *I* = 8, *D* = 0. The MIP-covered side of the aluminum chip faces the liquid reservoir of the flow cell, which is formed by an O ring to avoid leakage and to define a contact area of 28 mm^2^ with an internal volume of 110 μL. This setup is connected to syringe pumps that control the flow rate of the infusions, and therefore the concentration of the target molecule in the solution that comes into contact with the sensor. In particular, a flow rate of 0.125 mL min^–1^ was used with each of the seven injections lasting 5 min, followed by a stabilization period of 20 min. The temperature of the solution inside the flow cell (*T*_2_) was measured by another thermocouple placed 1 mm above the sensor (Figure 2).

Before every measurement, the flow cell was filled with PBS (without pyocyanin) and the signal was allowed to stabilize for 30 min, after which the concentration was gradually increased over a range of 1.4–9.8 μM. Rebinding of pyocyanin is expected to lead to a decrease in *T*_2_ at every stepwise increase in pyocyanin concentration, while *T*_1_ is kept constant at 37 °C. The HTM was used to study the sensitivity of the sensor toward pyocyanin and to analyze its selectivity toward the following molecules: l-ascorbic acid, riboflavin, glucose, and phenazine methosulfate, as well as the sensor’s selectivity toward pyocyanin in complex fluid media, such as King’s A medium and saliva.

### *Pseudomonas aeruginosa* Growth

Freeze-dried *P. aeruginosa* (DSM 50071-0121-001) were reactivated using the DSZM protocol, and half of the reactivated solution was stored in 30% glycerol at −20 °C. For every experiment, a new culture was started from the frozen stock into 20 mL of King’s A medium. A calibration curve was prepared with the colony counting method (detailed information about the calibration curve preparation can be found in the Supporting Information, Figure S4), and the obtained equation was used to quantify *P. aeruginosa* concentration for the analyzed samples.

### Indirect Detection of *P. aeruginosa* in King’s A Medium

The nutrient broth was prepared by dissolving proteose peptone (20 g), potassium sulfate (10 g), and magnesium chloride (1.64 g) in 1 L of deionized water, without adding the agar to keep the medium liquid. For every experiment, a new culture was started by suspending frozen bacteria in fresh nutrient broth. In particular, 20 mL of King’s A medium was inoculated with frozen *P. aeruginosa* and grown for 48 h at 30 °C with stirring set at 200 rpm. To assess if the sensor could detect the bacteria present in the medium by measuring pyocyanin, the incubation was stopped after 48 hours of growth. Next, 5 mL of the solution was withdrawn and diluted with sterile PBS solution in a 1:4 ratio to reach a final volume of 20 mL with a bacteria concentration of 9 × 10^8^ CFU mL^–1^. The amount of bacteria present in the sample was determined by the method described in the previous section. This solution was used to gradually expose the sensor to an increasing concentration of bacteria (and therefore pyocyanin) and the response of the sensor monitored.

### Saliva Samples Preparation

Saliva samples that did not contain any *P. aeruginosa* or pyocyanin were collected from one of the authors. The content was centrifuged twice at 5000 rpm for 10 min to remove any residual air from the solution. After centrifugation, the samples were spiked with pure pyocyanin to reach the desired concentration and used for HTM measurements with no further modification. To assess the sensor’s capability for the detection of pyocyanin in complex biological samples and therefore its potential application in medical diagnosis, it was exposed to gradually increasing concentrations of pyocyanin in saliva. In particular, following the centrifugation, 20 mL of saliva was spiked with 200 μL of a 140 μM solution of pyocyanin in PBS, reaching a final concentration of 1.4 μM.

## Results and Discussion

### Synthesis and Analysis of MIPs

**Chart 1:**
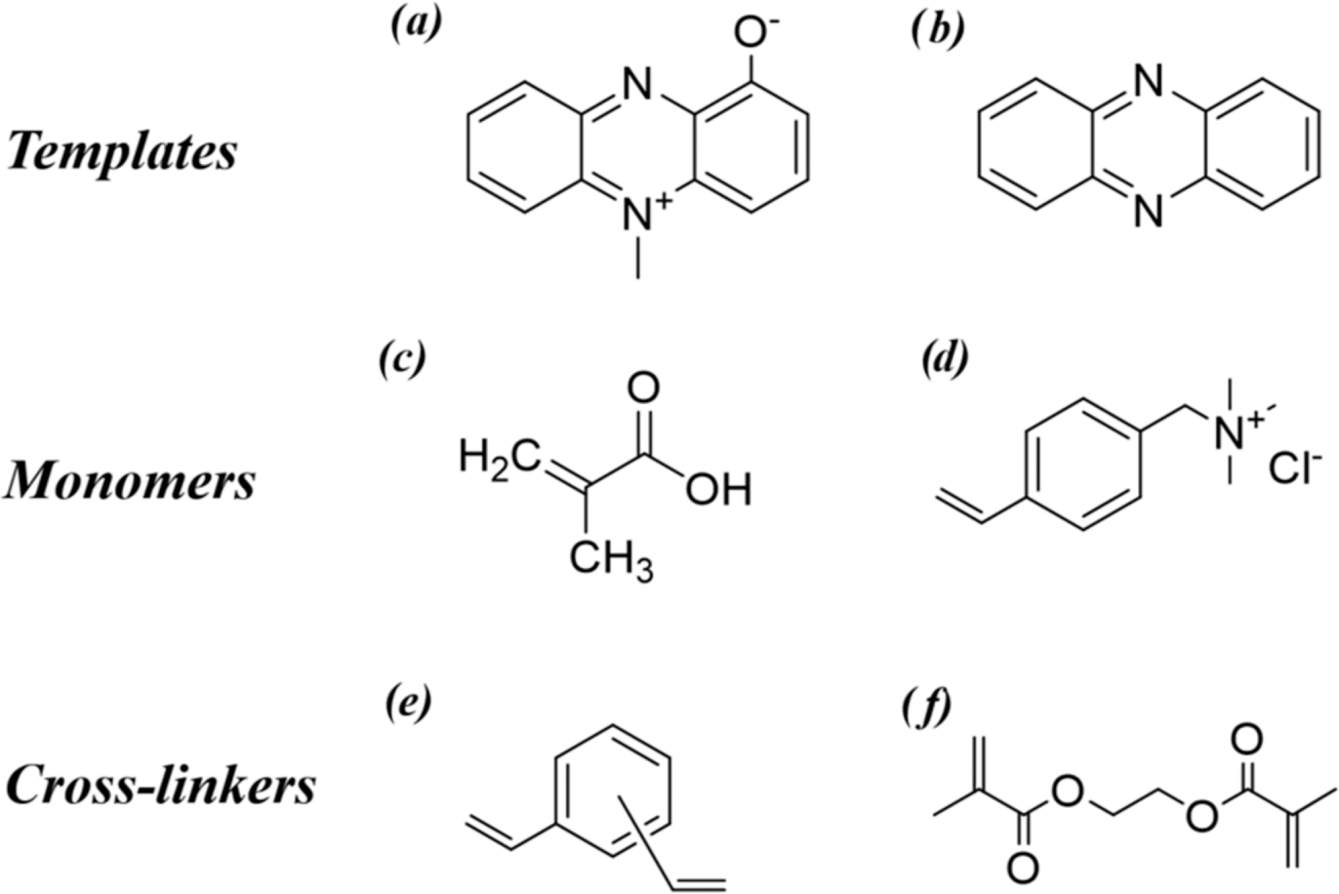
Chemical Structures of (a) Pyocyanin; (b) Phenazine; (c) Methacrylic Acid; (d) VBTMA; (e) Divinylbenzene; and (f) EGDMA

Due to the high radical scavenging properties of pyocyanin, an alternative dummy template molecule was selected to emulate the structure of pyocyanin but also to allow the free-radical polymerization process to take place. Architecturally, phenazine is a logical choice of dummy template as it has the same structural backbone as pyocyanin but lacks the alcohol functionality that is the source of the radical scavenging properties that impede polymerization (Chart 1). In addition, phenazine is highly soluble in organic media and has an absorption spectrum that is easily distinguishable from that of pyocyanin.^46^ This facilitates extraction of the template from the MIPs and enables interference-free UV–vis analysis of the generated MIPs.

Of the compositions analyzed for the binding of pyocyanin (Table 1), it is observed that polymer recipes containing VBTMA result in receptors with superior specificity compared to compositions containing MAA (Figure S5 in the Supporting Information shows the possible interactions between the monomers and the templates). Comparing the structures of these two monomers it is observed that VBTMA can provide two modes of interaction with the template (pi-stacking and ionic interactions), whereas MAA can only facilitate one (hydrogen bonding), thus yielding a lower level of affinity toward the pyocyanin, which provides a potential explanation for the improved imprinting factor observed in the resulting MIPs (Chart 1).

**Figure 3:**
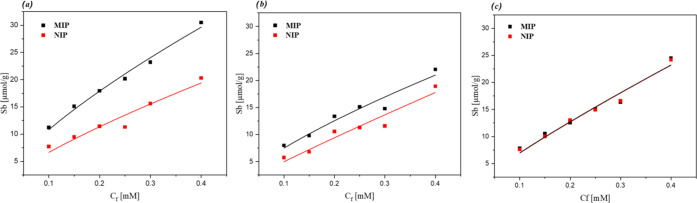
Binding isotherms for (a) MIP01, (b) MIP02, and (c) MIP03 after exposure to increasing concentrations of pyocyanin (0.1–0.4 mM) with MIP data represented as black squares and NIP data red squares.

A similar trend can be observed for the compositions containing EGDMA and DVB as functional cross-linkers. EGDMA promotes hydrogen bonding and produces hydrophilic polymer networks, while DVB is responsible for pi-stacking and produces hydrophobic polymers. The best rebinding results, performed in PBS buffer solutions, were obtained with EGDMA-cross-linked polymers, a finding that could be attributed to the hydrophilic nature of the resulting polymers and the resulting promotion of hydrogen bond formation in aqueous media. These qualitative observations are reflected in the gathered empirical data, as MIP04 (containing MAA and DVB) only has a binding capacity of 6 μmol g^–1^ in comparison to MIP01 (composed of VBTMA and EGDMA) having a binding capacity of 30 μmol g^–1^. Of the compositions summarized in Table 1, MIP01, 02, and 03 were carried forward for direct comparison in a batch-rebinding experiment, using a UV–vis spectrophotometer and analyzing the rebinding effect at a wavelength corresponding to 690 nm (λ_max_). These compositions contained the same constituent components but differed in stoichiometric ratios or solvent used. The binding isotherms obtained after the rebinding experiments for these MIPs were plotted (substrate bound (*S*_b_) vs free concentration (*C*_f_)) and allometrically fit (*y* = *ax*^*b*^) to emulate the saturation effects of the MIPs with increasing concentrations (Figure 3).

Among the MIPs that present an imprinting factor higher than 1, MIP01 is established to be the best-performing receptor, having the highest binding capacity (30 μmol g^–1^) and therefore greatest affinity toward the pyocyanin in comparison to the other MIPs. MIP02 shares the same stoichiometry as MIP01 but instead utilizes DMSO as a porogenic solvent rather than chloroform, indicating that the higher polarity solvent has a negative effect on the formation of the complementary binding cavities toward pyocyanin during the synthesis.

A more direct analysis of the sensor materials is possible by calculating the imprinting factor (IF) of each polymer (Table 1), being defined as the binding of the MIP divided by that of the NIP at a defined free concentration (*C*_f_). The NIP therefore acts as a negative control, facilitating a direct comparison between the MIP and enabling a standardized metric to be associated with the imprinting process (specificity of binding). For this calculation, a *C*_f_ of 0.15 mM was selected, with a lower concentration being preferable as it is less affected by the saturation effects observed at higher free concentrations. As a result, MIP01 is noted to have an IF = 1.59, whereas MIP02 has a diminished IF of 1.32 and MIP03 an IF of only 1. These results demonstrate that the composition of the polymer will dramatically influence the performance of the resulting MIP. This opens up the possibility of assessing other free-radical polymerization routes (e.g., polyurethanes, fluorescent monomers, grafting on magnetic cores, etc.). Therefore, MIP01 was selected as the optimal receptor and was used for further characterization by integrating it into the HTM-based sensing platform.

To study the saturation of the optimum receptor, an additional rebinding experiment was performed to fully understand the dynamic range of MIP/NIP01. To this end, the receptor was exposed to a wider concentration range (0.05–2 mM) of pyocyanin, and the saturation of the polymer was analyzed in an identical manner as conducted previously (see Figure S6 in the Supporting Information). The results further solidify the performance of the receptor, bolstering the calculated imprinting effect and demonstrating that the sensor can be exposed to a higher range of pyocyanin concentrations before succumbing to saturation effects.

### Heat-Transfer Method (HTM) Rebinding Experiments

**Figure 4:**
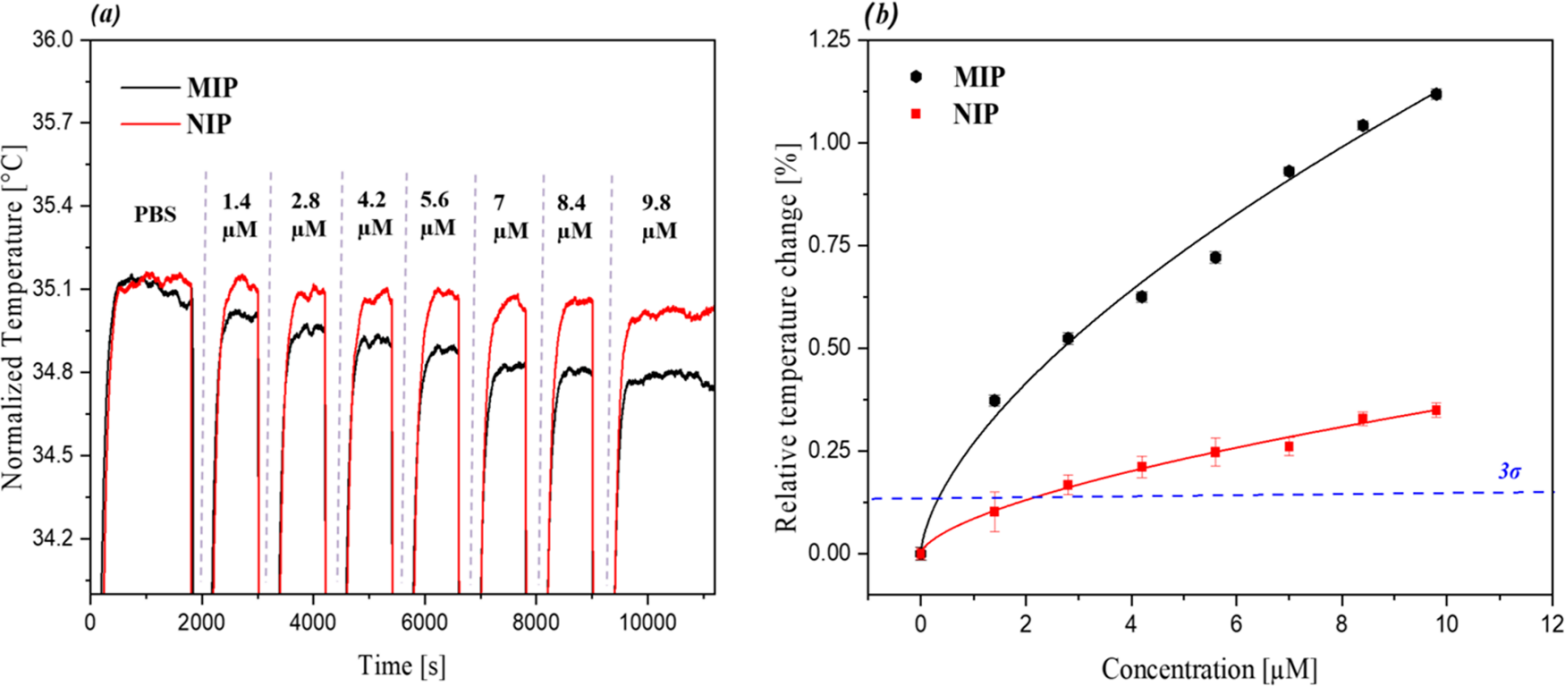
HTM rebinding analysis of MIP/NIP01. (a) Temperature profile over time of MIP (black) and NIP (red) upon exposure to increasing pyocyanin concentrations (1.4–9.8 μM) expressed as normalized temperatures; (b) extrapolated dose response with MIP (black squares) and NIP (red squares) fitted allometrically (MIP: black line—*R*^2^ = 0.978, NIP: red line—*R*^2^ = 0.983).

Once it was confirmed that MIP01 was the most effective MIP synthesized, the polymer was integrated into the thermal sensing platform and the rebinding performance was further studied with HTM analysis in PBS. All of the pyocyanin solutions were prepared in PBS to keep the pH at 7.4, thus ensuring the presence of the zwitterionic form of the toxin as this is the form associated with bacterial infections.^47^ All samples were prepared simultaneously to ensure higher reproducibility, with each analysis being performed in triplicate. During the analysis, the receptors were gradually exposed to increasing concentrations of pyocyanin (1.4–9.8 μM), with the temperature of the infused solution being continuously monitored (Figure 4a). With each new infusion of pyocyanin, a distinct decrease in the temperature of the stabilized solution inside the flow cell is observed, indicating that as pyocyanin binds to the receptor it impedes the flow of heat from the copper block to the solution inside the flow cell. This effect is most pronounced for the MIP (black line), with the temperature dropping 0.36 °C across the entirety of the experiment in comparison to the NIP (red line) that only drops by 0.09 °C. This is a logical observation, as the MIP is specifically tailored toward the specific binding of the pyocyanin and therefore binds a greater fraction of the compound, whereas the pyocyanin interaction with the NIP is nonspecific and the interaction less pronounced.

To further understand how the amount of pyocyanin that binds to the MIP layer correlates to a change in the monitored temperature, a dose–response graph was constructed for both MIP and NIP (Figure 4b). As MIP and NIP stabilized at different temperatures, the signal change was normalized and converted into a relative temperature change (%) for a more direct comparison (see eq 1). In the equation shown, Δ*T* is the change in temperature after infusion and *T*_PBS_ is the initial baseline stabilization temperature in PBS.
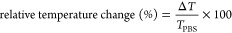
1

The plotted data was fit allometrically (*y* = *ax*^*b*^) accounting for the saturation effects observed by the receptors at high pyocyanin concentrations. This standardized comparison highlights the performance of the MIP, with the overall relative temperature change at 9.8 μM being 1.12% in comparison to the relative change of the NIP only being 0.30%. This indicates that the MIP has a significantly higher interaction with pyocyanin than the reference material. Moreover, the lower standard deviation witnessed by the MIP could stem from the specific interaction of the receptor with the pyocyanin resulting in a more stable interaction. This can be rationalized by the fact that the pyocyanin-NIP interaction should alternate more frequently between a bound and unbound state, thus leading to variations in the monitored temperature inside the flow cell. The concentration-dependent response of the MIP was used to calculate the limit of detection (LoD) for the sensor, using the widely accepted 3σ method.^48^ Using this methodology the LoD for the MIP was calculated to be 0.374 ± 0.027 μM. This illustrates that the sensor is able to operate in medically relevant concentration regimes, considering that is 20 times lower than the amount typically found in clinical samples of infected patients.^49−51^ To further investigate the sensor’s behavior and the saturation of the system, a more detailed study using a broader concentration range (1–80 μM) was performed (Figure S7 in the Supporting Information). The data from this experiment shows that the effect size linearly increases with concentration up to 30 μM, after which the sensor gradually levels off. This indicates that the sensor’s dynamic range spans two orders of magnitude in a diagnostically relevant concentration regime.

### Selectivity Measurements with HTM

**Figure 5:**
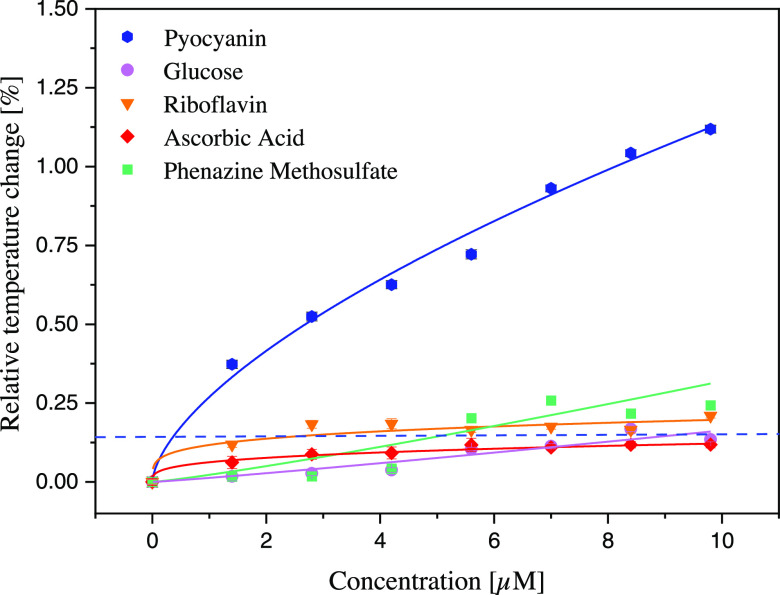
Dose–response graph extrapolated from the HTM analysis of glucose, ascorbic acid, riboflavin, and phenazine methosulfate toward MIP01 at varying concentrations.

To assess the selectivity of the sensor, four different molecules were chosen as analogues and the sensor’s reaction to these molecules was studied using HTM. The compounds chosen are the biologically relevant interferants l-ascorbic acid, riboflavin, and glucose and the chemical analogue phenazine methosulfate (see their chemical structure in the Supporting Information, Chart S1). The response to every molecule was studied in the same concentration range as pyocyanin (1.4–9.8 μM) to ensure an accurate comparison, and the aqueous solutions in PBS were prepared fresh prior to every measurement. As in previous experiments, the temperature inside the flow cell was monitored for each of the species introduced, with the normalized relative temperature change being calculated for each molecule (see Supporting Information, Figure S8 for raw data related to selectivity measurements). The result of this study demonstrates that the sensor is highly selective toward pyocyanin (Figure 5). The interaction with glucose and ascorbic acid is negligible with the sensor showing very little response when exposed to these molecules. Riboflavin and phenazine methosulfate induce a more prominent response but when comparing this effect to that of pyocyanin it can be noticed that it barely reaches the LoD threshold and would therefore not limit the performance of the sensor in complex samples. Overall, the response of the sensor toward the interferent molecules is comparable to that of pyocyanin with the reference NIP, thus highlighting the selectivity of the sensor even in the presence of a structural analogue, as was also previously demonstrated in prior research.^52^

### Proof-of-Principle Indirect Detection of *P. aeruginosa*

**Figure 6:**
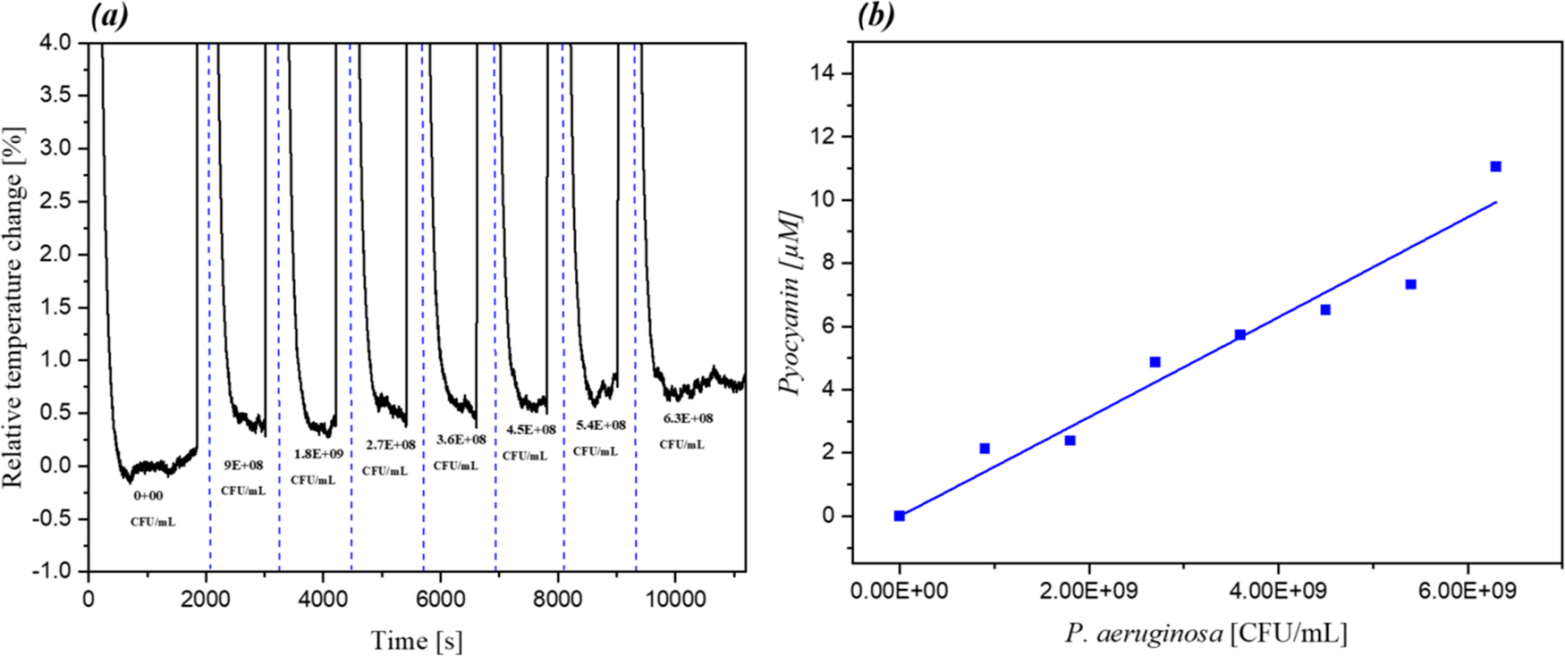
Dose–response graphs extrapolated from the HTM analysis of *P. aeruginosa* grown in King’s A medium toward MIP01 at varying concentrations. (a) Relative temperature change calculated from the temperature profile using eq 1; (b) correlation between pyocyanin and bacteria concentration.

To determine if the developed sensor could indirectly detect the presence of *P. aeruginosa* by sensing pyocyanin, an experiment was devised where the receptor was exposed to bacteria cultures grown in King’s A medium. HTM studies were conducted using the same setup as described for the previous measurements, and the sensor was exposed to complex solutions containing increasing concentrations of *P. aeruginosa*. The obtained temperature profile data were averaged and converted into a relative temperature change using eq 1 (Figure 6a). The data shows that the sensor response increases proportionally to the amount of bacteria present in the culture medium. The amount of pyocyanin measured in the sample (*y*-axis) can be directly correlated to the amount of bacteria present in the culture sample (Figure 6b). The HTM results were benchmarked by extracting the bacteria using a 1:1 ratio of King’s A and chloroform, which is then reextracted into an equal volume of HCl 0.2M following the procedure.^53^ The obtained solution was diluted 1:4 in HCl 0.2M to obtain the same concentration used for the measurements, then analyzed using a UV–vis spectrophotometer at 520 nm. The resulting absorbance corresponds to a concentration of 1.67 μM, proving that the real pyocyanin concentration inside the sample is in line with the HTM results. This proof of principle demonstrates that the developed sensor represents a first stage of a low-cost, rapid test that could be used to confirm the presence of a *P. aeruginosa* infection in complex fluids without the need for sample pretreatment.

### Pyocyanin Detection in Saliva

**Figure 7:**
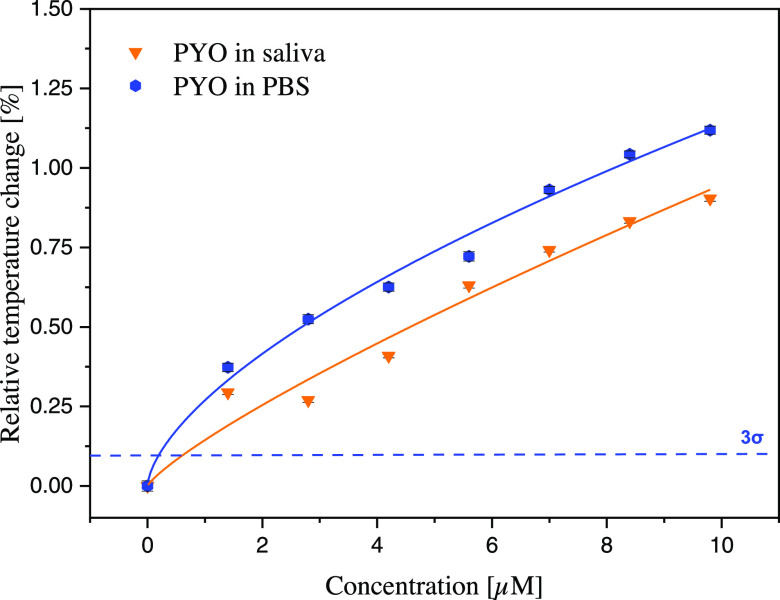
Dose–response graph extrapolated from the HTM analysis of pure pyocyanin in PBS (blue curve) and saliva spiked with pyocyanin (orange curve).

To further demonstrate the use of the sensor in complex samples and illustrate its applicability in medical diagnostics, a study in a simulated clinical sample was performed. It is known that the pyocyanin concentration in the airways can be up to 100 μM during infections and pyocyanin is therefore used in clinical diagnostics as a biomarker for the fast diagnosis of a *P. aeruginosa* infection for immunocompromised persons such as cystic fibrosis patients.^6^ Therefore, saliva samples were collected and spiked with pure pyocyanin (see Section Saliva Samples Preparation) and used to perform HTM analysis in the same concentration range as per previous measurements (1.4–9.8 μM), so as to assess the sensor response to medically relevant concentration. As a negative control, a measurement in a nonspiked sample was performed and compared to the spiked samples (Figure 7).

The results summarized in Figure 7 clearly show that the sensor can detect pyocyanin inside saliva successfully (orange line), with the complexity of the simulated clinical sample only marginally decreasing the observed effect size at each concentration (blue line). This measurement proves the feasibility of a real-life application for hospitalized patients, offering the possibility to detect an ongoing infection by analyzing sputum samples with a limit of detection of 0.569 ± 0.063 μM. The results of this study are promising, primarily considering that the pyocyanin concentration detected in hospitalized patients is at least 10 times higher than the limit of detection hereby calculated,^5^ and considering that the LoD obtained is lower than other sensing technologies developed for a similar purpose.^54,55^ As the concentration of pyocyanin in saliva samples of CF patients where a *P. aeruginosa* infection is not present, will be virtually zero, the sensor seems to be excellently suited to provide practitioners with a 1/0 decision tool when analyzing saliva samples of suspected patients.

## Conclusions

The work presented in this paper illustrates the use of a thermal MIP-based sensor for the indirect detection of *P. aeruginosa* through pyocyanin, the main toxin excreted by the bacterium. The study shows that it is possible to create and optimize a MIP for the detection of pyocyanin using phenazine as a dummy template. The resulting MIP has shown to be selective toward a pyocyanin solution in a concentration range of 1.4–9.8 μM, a range specially chosen to mimic the lowest pyocyanin concentration found in real samples, such as saliva, sputum, or urine. The specificity and sensitivity of the MIP were analyzed by comparing the sensor’s response toward potential interferents encountered in biological samples and by studying the response of a NIP-coated chip as a negative control. As a first proof-of-application, the sensor was used to detect the presence of the toxin in a nutrient broth (King’s A medium) also containing *P. aeruginosa*, demonstrating that the sensor is able to pick up pyocyanin shed by bacteria growing in culture, confirming in this way the hypothesis of the research. To further assess the usability of the sensor in complex media, a similar study was performed in spiked saliva samples, indicating that the sensor is capable of detecting pyocyanin, as a marker of *P. aeruginosa* infection, at clinically relevant concentrations (LoD of 0.569 ± 0.063 μM). This highlights the sensor’s potential for application in fast tests for the detection of infection in immunocompromised patients. Therefore, possible follow-up research should be aimed at creating a more thorough understanding of the sensor’s performance in a wide array of clinical and biological samples and further exploring the limits of the technology.

## Supporting Information Available

Chemical structures of phenazine methosulfate, glucose, l-ascorbic acid, and riboflavin (Chart S1); HPLC chromatogram of pyocyanin purification (Figure S1); gradient used for HPLC purification (Table S1); LC-MS chromatogram of purified pyocyanin (Figure S2); ^1^H NMR (400 MHz, DMSO-D6) of synthesized pyocyanin (Figure S3); procedure for the calibration curve preparation for *P. aeruginosa* (Figure S4); schematic representation of the interactions between monomers and templates (Figure S5); binding isotherms for MIP01 and NIP01 after exposure to increasing concentrations of pyocyanin (0.05–2 mM) (Figure S6); HTM measurements for MIP01 and NIP01 upon exposure to increasing pyocyanin concentrations (1–80 μM) (Figure S7); raw data of HTM selectivity measurements (Figure S8); and raw data of HTM temperature profiles of pyocyanin in King’s A medium containing *P. aeruginosa* (Figure S9) (PDF)

## Abbreviations

PYO: pyocyanin
MIP: molecularly imprinted polymer
NIP: non-imprinted polymer
HTM: heat-transfer method
IF: imprinting factor
DVB: divinylbenzene
PVC: poly(vinyl chloride)
PDMS: poly(dimethylsiloxane)
LoD: limit of detection
*S*_b_: substrate bound
*C*_f_: final concentration
