# Supplementary material for: Thermal Pyocyanin Sensor Based on Molecularly Imprinted Polymers for the Indirect Detection of Pseudomonas aeruginosa
Source: ACS Sens. 2023 Jan 4;8(1):353–62. doi: 10.1021/acssensors.2c02345 (PMC9887650; doi:10.1021/acssensors.2c02345)
Supplement: Supplementary file 1 — se2c02345_si_001.pdf [file se2c02345_si_001.pdf]

Thermal pyocyanin sensor based on molecularly imprinted polymers for the indirect detection of *Pseudomonas aeruginosa*

-

Supporting Information

-

Margaux Frigoli[a*], Joseph W. Lowdon[a], Manlio Caldara[a], Rocio Arreguin-Campos[a], Julia Sewall[a] ,Thomas J. Cleij [a], Hanne Diliën [a], Kasper Eersels [a], Bart van Grinsven [a]

[a] Sensor Engineering Department, Faculty of Science and Engineering, Maastricht University, P.O. Box 616, 6200 MD Maastricht, the Netherlands

* Corresponding author: m.frigoli@maastrichtuniversity.nl

- Supplementary **Chart S1**

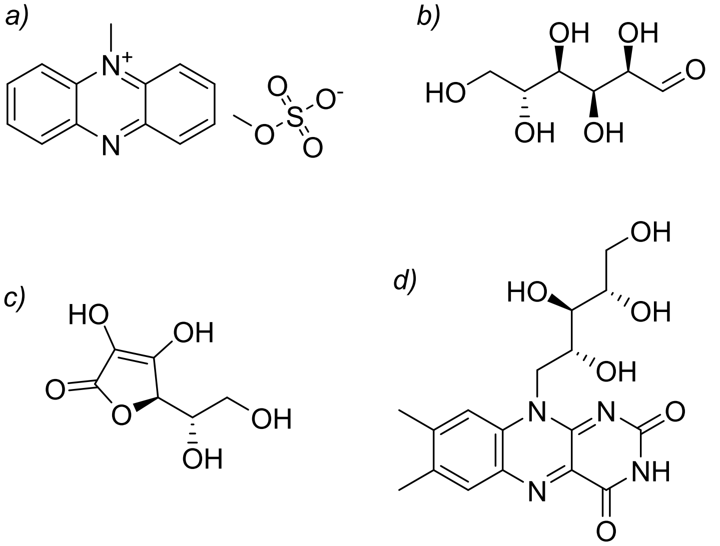

Chart S1: *Chemical structures of a) phenazine methosulfate; b) glucose; c) L-ascorbic acid; d) riboflavin.*

- Supplementary **Figure S1**

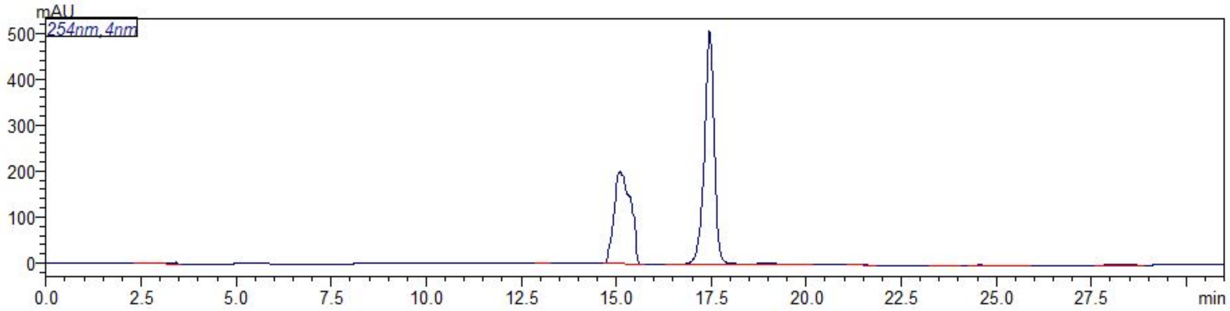

Figure S1*: HPLC chromatogram of pyocyanin purification. Product retention time is 17.5 min.*

High-pressure Liquid Chromatography

In order to achieve pure pyocyanin, a Shimadzu LC-20AR HPLC system was used. The instrument was equipped with a Shim-Pack Prep-ODS-column with 15u particle size, internal diameter 30 mm, length 250 mm. The solvents used were water + 0.1% of acetic acid and methanol + 0.1% of acetic acid, all purchased from Sigma Aldrich as HPLC grade solvents.

- Supplementary **Table S1**

Table S1: *Gradient used for HPLC purification. Solvent A is water + 0.1% of acetic acid, solvent B is methanol + 0.1% of acetic acid.*

**Time [min]**	**Flow [mL/min]**	**A. Conc**	**B. Conc**
1.00	1.000	95.0	5.0
2.00	15.000	90.5	9.5
21.00	15.000	5.0	95.0

21.10	20.000	5.0	95.0
23.50	20.000	5.0	95.0
25.00	20.000	95.0	5.0
26.00	20.000	95.0	5.0
27.00	1.000	95.0	5.0

- Supplementary **Figure S2**

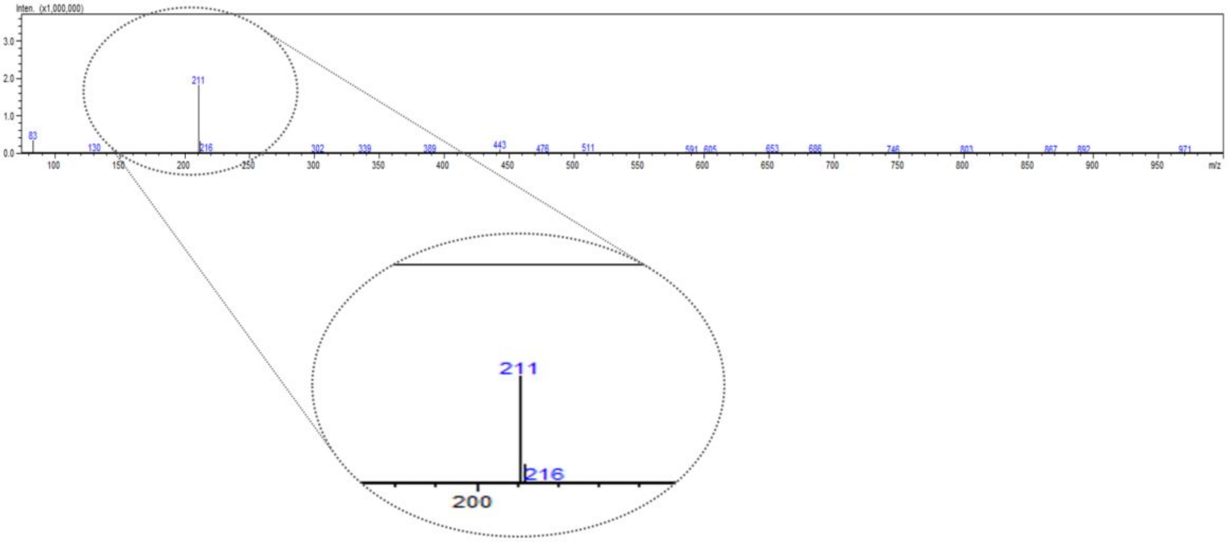

Figure S2: *LC-MS chromatogram of purified pyocyanin. Exact Mass* $[M+H]^{+}$ *: 211.23.*

- Supplementary **Figure S3**

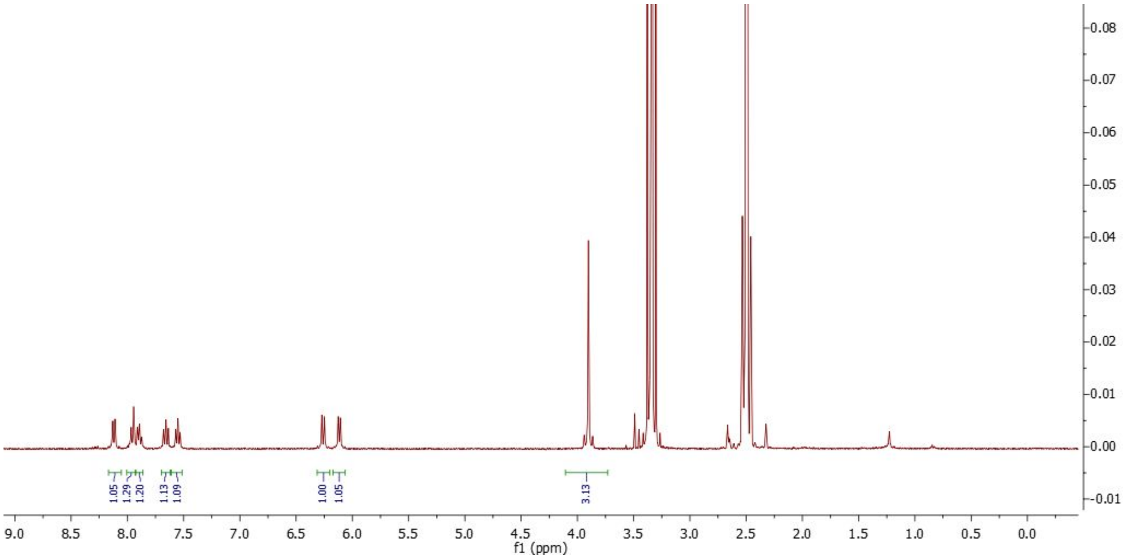

Figure S3*:* ^{1}H *NMR (400 MHz, DMSO-*D_6*) of synthesized pyocyanin. δ 8.13, 8.11, 7.97, 7.97, 7.94, 7.91, 7.89, 7.87, 7.68, 7.66, 7.64, 7.57, 7.55, 7.53, 6.27, 6.25, 6.13, 6.11, 3.94, 3.90, 3.86, 3.49, 3.38, 3.34, 3.30, 2.65, 2.54, 2.54, 2.53, 2.53, 2.52, 2.51, 2.50, 2.50, 2.49, 2.49, 2.47, 2.46, 2.46, 2.45, 2.45, 1.23.*

- **Procedure for the calibration curve preparation for *P. aeruginosa*:**

1. A new culture in 20mL of King`s A medium was started from a frozen stock of *Pseudomonas aeruginosa*, and the solution was grown for 24h at 30 ℃ stirring at 200 rpm.
2. The liquid medium was measured with a UV-Vis spectrophotometer. The obtained OD_{660} was 1.088.
3. Starting from the OD_{660} = 1.088, six dilutions were performed with the chosen medium to reach different OD_{660} (0.1, 0.2, 0.3, 0.4, 0.5, 0.6).
4. For every OD660 chosen, five dilutions were performed and 0.1 mL of each dilution were plated onto agar plates for 24h (**Figure S4**).
5. The resulting colonies were counted and converted into CFU/mL, then a calibration curve was obtained by plotting the CFU/mL vs OD_{660}. It was found that OD660 = 1 corresponds to $2.97 \cdot 10^9\ CFU/mL$.

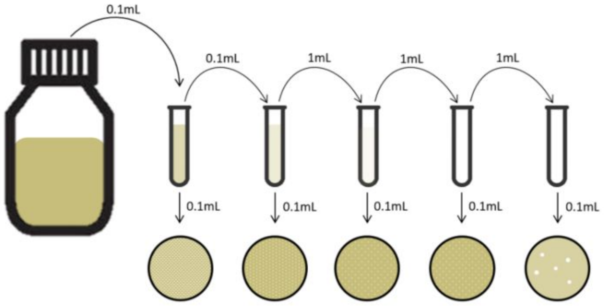

***Figure S4**: Dilutions performed for the calibration curve of Pseudomonas aeruginosa.*

- Supplementary **Figure S5**

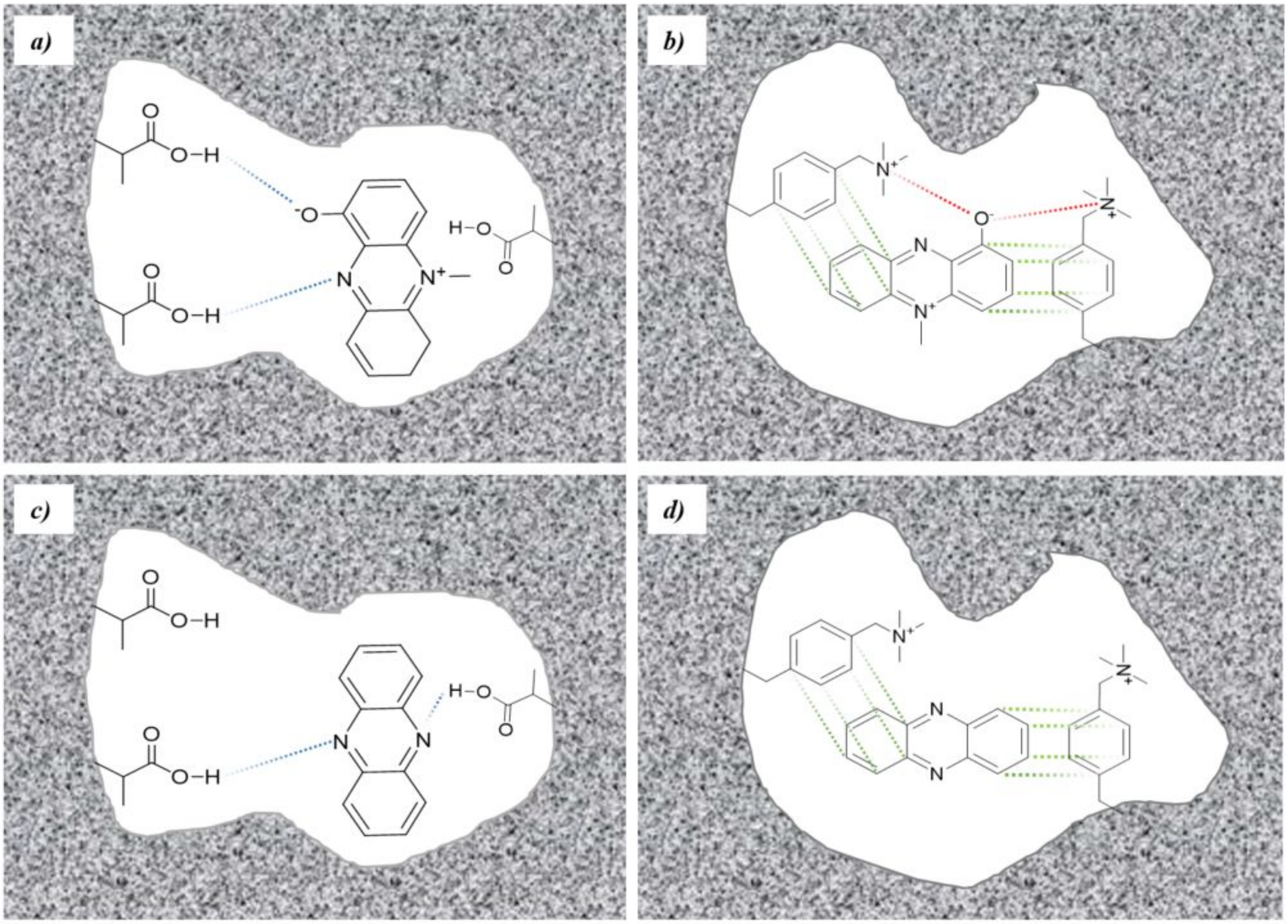

Figure S5: *Schematic representation of the interactions between monomers and templates: a) MAA and pyocyanin; b) VBTMA and pyocyanin; c) MAA and phenazine and d) VBTMA and phenazine. Ionic interactions are drawn in red, pi stacking in green and H bonding in blue.*

- Supplementary **Figure S6**

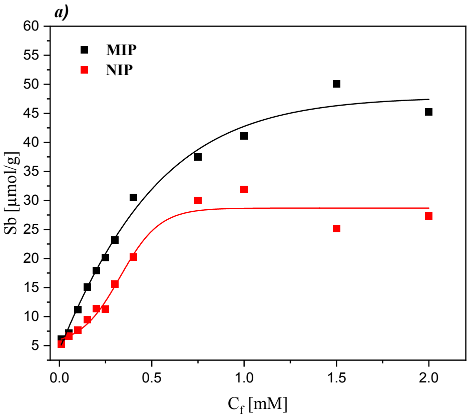

Figure S6: *Binding isotherms for MIP01 and NIP01 after exposure to increasing concentrations of pyocyanin (0.05 – 2 mM) with MIP data represented as black squares and NIP data red squares. Collected data were fitted using a dose-response fitting method obtaining R2 values of 0.999 (MIP01) and 0.957 (NIP01).*

- Supplementary **Figure S7, S8, S9**

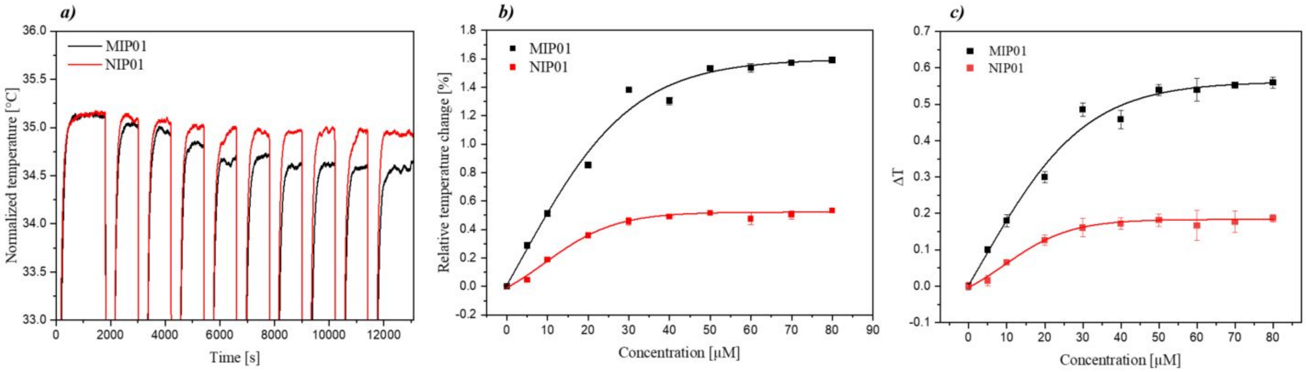

Figure S7: *HTM measurements for MIP01 (black line) and NIP01 (red line) upon exposure to increasing pyocyanin concentrations (1-80 µM). a) temperature profiles; b) relative temperature change in percentage and c) temperature difference obtained from the temperature profiles.*

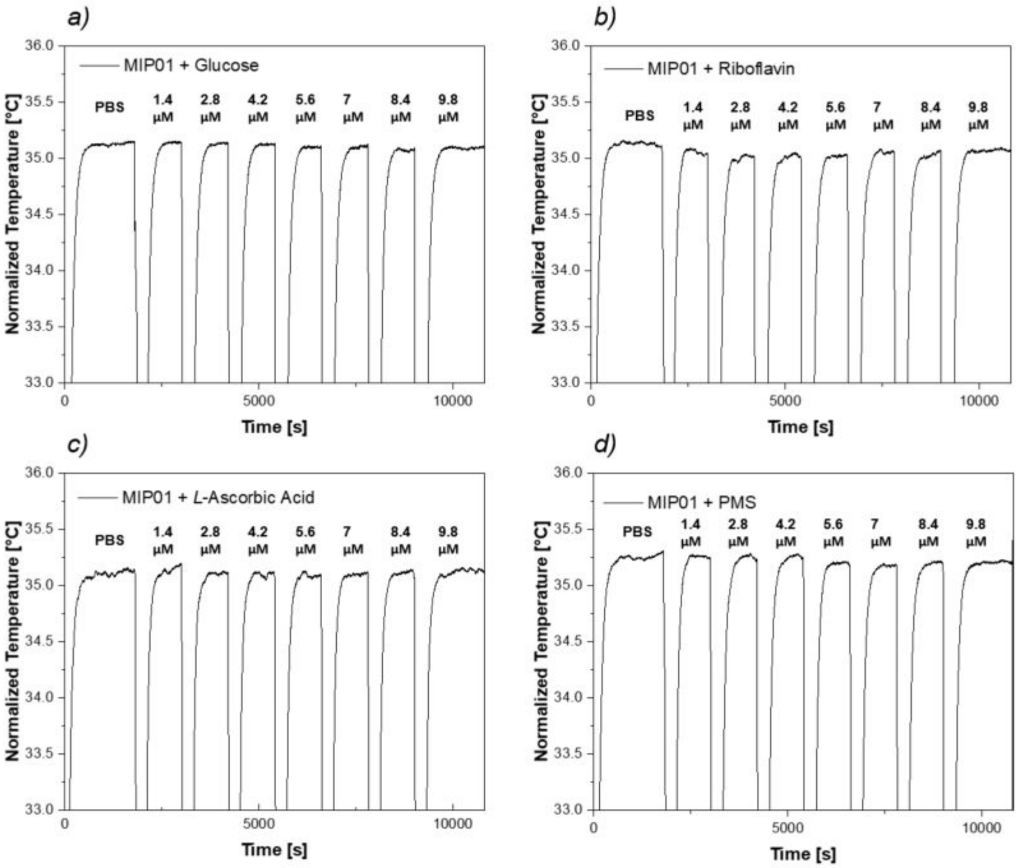

Figure S8: *Raw data of HTM selectivity measurements (performed in triplicate). a) glucose; b) riboflavin; c) L-ascorbic acid; d) Phenazine methosulfate.*

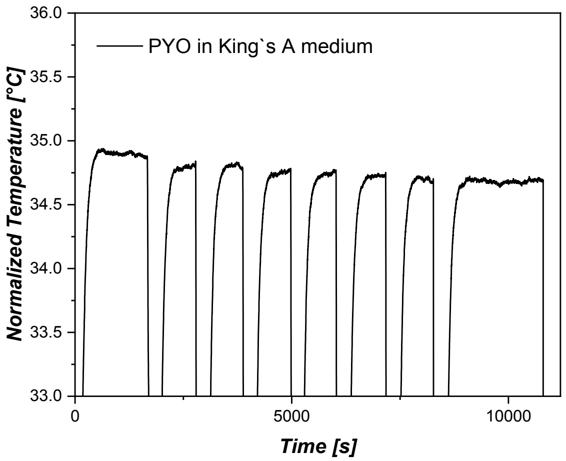

Figure S9: *Raw data of HTM temperature profiles (performed in triplicate) of pyocyanin in King`s A medium containing Pseudomonas aeruginosa.*
